# Invasive Fungal Infections Following Kidney Transplantation

**Published:** 2012-08-01

**Authors:** B. Einollahi

**Affiliations:** *Nephrology and Urology Research Center, Baqiyatallah University of Medical Sciences, Tehran, Iran*

**DEAR EDITOR,**

 I read with great interest an article of Ezzatzadegan, *et al*, recently published in *IJOTM* [1]. They reported their experience about the incidence and etiology of invasive fungal infections (IFIs) among 471 kidney transplant recipients within a decade.

I agree that the recent reports show a decline in the number of IFIs after kidney transplantation; however, IFIs are associated with high mortality after solid organ transplantation [2]. In Transplant-Associated Infection Surveillance Network database, there were 1063 confirmed and probable cases of IFI in solid organ transplant recipients [3]. The overall incidence of IFIs varies according to the type of transplant; it is lowest in kidney transplant patients when compared to other solid organ recipients [3]. On the other hand, considering the growing number of immunocompromised kidney recipients, the incidence of IFIs may be increased after renal transplantation; due to its high mortality, soon it may be an important concern among these patients. Furthermore, the prevalence of IFIs following kidney transplantation in Iran (0.9%) is the lowest rate among the Middle East countries; the prevalence in Turkey, Kuwait and India is 4%, 3.5% and 14%, respectively [2].

On the contrary to what Ezzatzadegan, *et al*, reported that *Cryptococcus neoformans *accounted for 50% of all IFIs [1], we found that *Aspergillus* and *Candida* were responsible for more than 80% of IFIs in organ transplant recipients [2]. In 2410 studied kidney transplants, mucormycosis accounted for 52% of all invasive mycoses; however, mucormycosis with an incidence of 0.2%–1.2% per year, represented a small portion of IFIs in kidney transplants [2]; it is in contrary to what Ezzatzadegan, *et al*, found—they reported only one case of mucormycosis in their kidney transplant patients [1].

I appreciate that advanced age of recipients is a risk factor for developing IFI, as noted by Ezzatzadegan and colleagues [1], but in a large retrospective study on fungal infection in kidney transplant recipients, it was found that age >40 years was a risk factor for invasive fungal infection with mucormycosis [4].

According to Ezzatzadegan, *et al* [1], the mean time to develop IFI was 33 months after transplantation with majority of the infection occurred within the first 24 months. However, the occurrence of IFIs is highest in the first six months of transplantation when immunosuppression was most intense [3]. In our recipients, IFIs were more likely to occur within 12 months after renal transplantation [2]. In addition, the incidence of mucormycosis was 44%–59% within the first year of kidney transplantation [4,5].
